# Bonding, Bridging, and Linking Social Capital and Self-Rated Health among Chinese Adults: Use of the Anchoring Vignettes Technique

**DOI:** 10.1371/journal.pone.0142300

**Published:** 2015-11-16

**Authors:** He Chen, Tianguang Meng

**Affiliations:** 1 Department of Global Health, School of Public Health, Peking University, Beijing, China; 2 Department of Political Science, School of Social Sciences, Tsinghua University, Beijing, China; University of South Carolina, UNITED STATES

## Abstract

Three main opposing camps exist over how social capital relates to population health, namely the social support perspective, the inequality thesis, and the political economy approach. The distinction among bonding, bridging, and linking social capital probably helps close the debates between these three camps, which is rarely investigated in existing literatures. Moreover, although self-rated health is a frequently used health indicator in studies on the relationship between social capital and health, the interpersonal incomparability of this measure has been largely neglected. This study has two main objectives. Firstly, we aim to investigate the relationship between bonding, bridging, and linking social capital and self-rated health among Chinese adults. Secondly, we aim to improve the interpersonal comparability in self-rated health measurement. We use data from a nationally representative survey in China. Self-rated health was adjusted using the anchoring vignettes technique to improve comparability. Two-level ordinal logistic regression was performed to model the association between social capital and self-rated health at both individual and community levels. The interaction between residence and social capital was included to examine urban/rural disparities in the relationship. We found that most social capital indicators had a significant relationship with adjusted self-rated health of Chinese adults, but the relationships were mixed. Individual-level bonding, linking social capital, and community-level bridging social capital were positively related with health. Significant urban/rural disparities appeared in the association between community-level bonding, linking social capital, and adjusted self-rated health. For example, people living in communities with higher bonding social capital tended to report poorer adjusted self-rated health in urban areas, but the opposite tendency held for rural areas. Furthermore, the comparison between multivariate analyses results before and after the anchoring vignettes adjustment showed that the relationship between community-level social capital and self-rated health might be distorted if comparability problems are not addressed. In conclusion, the framework of bonding, bridging, and linking social capital helps us better understand the mechanism between social capital and self-rated health. Cultural and socioeconomic factors should be considered when designing health intervention policies using social capital. Moreover, we recommend that more studies improve the comparability of self-rated health by using the anchoring vignettes technique.

## Introduction

The concept of social capital (SC) has been used frequently to explain the disparities in population health in the last two decades. Scholars define social capital in different ways. For example, according to Coleman, “Social capital is defined by its function. It is not a single entity but a variety of different entities, with two elements in common: they all consist of some aspect of social structures and they facilitate certain actions of actors-whether persons or corporate actors-within the structure” (page 98 in [1]); Putnam defines social capital as “features of social organization such as networks, norms, and social trust that facilitate co-ordination and co-operation for mutual benefit” (page 67 in [2]). Although no agreement has been achieved on how to define social capital, most definitions include three elements, i.e. social network, norms of reciprocity and trust [3]. Moreover, the SC measurement is very challenging, due to the controversies around the SC definition and the difficulties in operationalizing SC variables such as trust and norm of reciprocity. In recent years, some studies have been conducted to develop a SC measurement scale, such as the Social Capital Assessment Tool (SCAT) by the World Bank’s social capital initiative [4]. Based on a comprehensive review of the SC measurement in relation to health in low and middle income countries including China, Agampodi *et al*. [5] recommended three scales, the Adapted SCAT by Harpham *et al*. [6], the six item tool by Hurtado *et al*. [7] and the World Values Survey Social Capital Scale by Elgar *et al*. [8]. Regretfully, the distinction between bonding, bridging, and linking SC that we will discuss in the following paragraphs is not satisfactorily reflected in these scales. Until now, there has not been a universally accepted gold standard tool for SC measurement [5].

There is a large body of literature indicating that SC matters for health (e.g., self-rated health [SRH] [9, 10], mental health [11, 12] and mortality [13, 14]). However, debates still exist over how SC relates to population health [15]. There are three main opposing camps. The social support perspective regards social support as the mechanism linking SC and health outcomes [16–18]. The inequality thesis argues that widening economic and social inequality causes anxiety among disadvantaged groups and increases disrespect and isolation between people with different social identities, influencing health through a socio-psycho-physiological mechanism [19–21]. The political economy approach posits that health inequalities are fundamentally the results of deficiencies in material resources and that the discussion of SC is only meaningful to population health within the context of society’s economic and political structures [22–24].

To reconcile these perspectives, Szreter and Woolcock expanded the theoretical framework of SC by distinguishing among bonding, bridging, and linking SC [15]. Bonding SC comprises relations of trust and cooperation among people with similar social identity (e.g., age, ethnicity, class); bridging SC refers to relations of respect and mutuality among people unlike in social identity but more or less equal in their status or power; linking SC is defined as “norms of respect and networks of trusting relationships between people who are interacting across explicit, formal or institutionalized power or authority gradients in society” (page 655 in [15]). Social support may promote health through either emotional (e.g. provision of empathy and caring), instrumental (e.g. help with money or jobs), or informational (e.g. provision of health-related information) support, or through social companionship (e.g. spending leisure time together) [3]. Although social support possibly comes from any of the three forms of SC, bonding SC is the main source [18]. On the other hand, in Wilkinson’s perspective, steep or widening social inequality in societies tends to increase anxiety, disrespect, and distrust between people, which compromises bridging SC [19]. In turn, the socio-psycho-physiological mechanism triggered along with low bridging SC undermines population health, in particular among those who perceive themselves in disadvantaged positions [19, 20]. Moreover, linking SC brings in the “state-society” relations, whose importance is emphasized by the political economy or “neo-material” approach. Lynch and his collaborators argue that even in affluent societies, some people still lack access to materials beneficial to their health such as healthy food [22, 25]. Such material deficiency is fundamentally associated with the political and ideological decisions on the relationship between state and citizens, which is captured by linking SC measuring the relationship between public service providers (e.g. officials and doctors) and citizens [15].

The bonding/bridging/linking SC framework has significant theoretical and practical implications. First, as we have illustrated above, it helps to resolve the debates between social support, inequality, and political economy positions. Because existing literatures indicate that all the three positions have a point [16, 19, 22], it is reasonable to include all of them in the theoretical framework of SC, instead of choosing one over another. In this perspective, the extended SC framework proposed by Szreter and Woolcock is more sophisticated and complete than others [26]. Second, it helps to improve the health interventions designed with social capital. According to the extended framework, the interventions only considering bonding and bridging SC probably fail to fulfill their objectives, and attention should also be paid to the relationships between people across power gradients [22, 23]. Measuring the stock of all three forms of SC and understanding their associations with health will provide a more comprehensive evidence basis for health interventions. Despite its advantages, the bonding/bridging/linking SC framework has been rarely used, probably due to lack of available data [26], and led to mixed conclusions, which impedes the theoretical development and practical application of SC [18, 27–29]. Poortinga indicated that most bonding, bridging, and linking SC indicators were positively related to SRH [18]. Bonding and linking SC also had health-promoting effects among Norwegian adults, yet bridging SC (measured as the ethnic diversity of one’s social network) was negatively associated with SRH [29]. Goryakin *et al*. [27] used five indicators to examine the influence of different forms of SC on self-rated general and mental health in nine former Soviet countries. Being trustful of the government increased the probability of reporting good general and mental health, and worries about being harassed or threatened on the street reduced it [27]. More studies are needed to examine the utility of the bonding/bridging/linking SC distinction for understanding population health.

Previous study indicated that social capital has different distributions in urban and rural China [30]. Currently, only two studies have compared the association between SC and health for urban and rural Chinese. Both studies showed urban-rural disparities in such relationships. In a study of elderly residents, Norstrand and Xu [28] found that in urban China, bonding SC, measured as trust in and a feeling of closeness to family members, friends, and neighbors, was beneficial for physical and emotional health, and linking SC, measured as number of years as a Communist Party member, was positively associated with physical health. However, there was no significant relationship between SC and physical/emotional health among rural elders [28]. In addition, our last study showed that individual-level bridging trust was positively related to SRH in urban areas, but not in rural areas [31]. Such disparities probably derive from the different cultural backgrounds and social relationships in urban and rural China [28, 31]. Without taking account of such differences, we might not be able to arrive at correct conclusions about the relationship between SC and SRH among Chinese.

SRH is a frequently used health indicator [26]; however, reporting bias may affect judgments of the relationship between SC and health. People’s assessment of their health status is influenced by many factors, such as culture, education, and knowledge of health, and thus survey results obtained from individuals or groups are not completely comparable [32]. The issue of incomparability might be especially important for our study since factors such as culture and education are often homogeneous within a single geographic area [33, 34]. Furthermore, SC is usually regarded as both an individual and contextual factor for health [10, 26]. Thus, it is possible that the association between SC and health is distorted, especially at the contextual level. Although multiple techniques have been developed to improve the comparability of response scales across groups, the anchoring vignettes technique proposed by King et al. [35, 36] is the most promising approach [37]. In the context of SRH, this technique uses vignettes (i.e., supplemental survey questions) to describe hypothetical people with fixed health traits. The variation in responses to the same vignettes provides information on differential item functioning (DIF) that is used to anchor individuals’ assessments of their own health [36]. This technique has been increasingly used in population health studies [37, 38], but has not been used in research on SC and health.

Using nationally representative data, we aim to investigate the relationship between bonding/bridging/linking SC and SRH among Chinese adults. In addition, by using the anchoring vignettes technique, we aim to improve the interpersonal comparability of SRH and further assess its influence on the association between SC and SRH.

## Methods

### Data source

Data came from the 2012 wave of China Family Panel Studies (CFPS), a longitudinal survey of Chinese communities, families, and individuals [39]. Follow-up surveys were conducted annually or biennially. Using an implicit stratification, multistage, probability proportional to size sampling technique, the 2010 baseline survey was conducted in 25 province-level administrative units (excluding Hong Kong, Macao, Taiwan, Xinjiang, Tibet, Qinghai, Inner Mongolia, Ningxia, and Hainan) and covered 95% of the population of mainland China. The CFPS 2010 interviewed 14,960 households from 635 communities, including 33,600 adults and 8,990 children. The response rate was 81.28% at the household level and 84.14% at the individual level. The CFPS 2010 data was evaluated with respect to distribution of age, sex, rural/urban residence, education, marital status, household type, household size, and income. The results showed that the CFPS 2010 was nationally representative [40].

All family members in 2010 baseline survey and their newborn/adopted children thereafter are defined as CFPS gene members and the CFPS intends to follow the progress of these individuals throughout their lives. All households including gene members will be interviewed until no gene member lives there, i.e. all gene members in the household have moved out or deceased [41]. The households interviewed in 2012 wave could be those interviewed in 2010, but they could also be new households which appear due to the marriages, divorces, or separations of gene members. CFPS 2012 interviewed a total of 13,453 households including 12,725 households from CFPS 2010 and 728 new households. The successful-tracking rate of CFPS 2012 was 85.1% at the household level and 80.6% at the individual level. Considering the sample attrition at both household and individual levels, the data representativeness of CFPS 2012 is probably negatively influenced to some extent [42].

Items on SC were not included in CFPS 2010 questionnaires, and thus we used CFPS 2012 data as cross-sectional data. In this study, we focused on persons aged 18 years and older. The final sample consisted of 22,940 individuals living in 411 communities in 107 county-level units. The sample size for each community ranged from 11 to 134.

### Measures

#### Self-rated health

We used the anchoring vignettes technique to measure SRH. According to King, Murray, Salomon, and Tandon [35], two assumptions were made: (1) *response consistency*, which assumes that each respondent uses the survey response categories in the same way to answer the anchoring vignettes and self-assessment questions; and (2) *vignette equivalence*, which assumes that the level of the variable represented in the vignette is understood by all respondents in the same way apart from random assessment error. Respondents assessed their health using a 5-point Likert scale (1 = excellent, 2 = very good, 3 = good, 4 = fair, 5 = poor). Then each respondent received two vignettes that described persons with two different health statuses (Table 1). Respondents answered the vignette questions using the same standard by which they had assessed their own health. Because the health traits described in the vignettes were fixed, differences among respondents for the same vignette reflected DIF. Based on this information on DIF, we recoded the SRH assessment using a nonparametric estimator (Table 2), which produced adjusted (or DIF-corrected) SRH with five ordinal categories, 1 indicating excellent SRH and 5 indicating poor SRH.

**Table 1:** Text of self-rated health and vignettes questions and response options.

Category	Content
**Self-rated health**	How would you rate your health status? Options: 1 = excellent, 2 = very good, 3 = good, 4 = fair, 5 = poor.
**Introductory text to Vignettes**	Now I am going to describe some persons who have health problems to different extents. I want to know how you would rate their health status according to the same standard you use to rate your own health status. Please imagine these persons have the same age and background as you.
**Vignette 1**	Jun Sun/Mei Li has no problems whenwalking, running, and moving limbs. He/she goes for a five-mile jog twice per week. He/she cannot remember the last time of he/she felt pain, because he/she has not felt pain in the last year, even after manual labor and physical exercise. How would rate the health status of Jun Sun/Mei Li? Options: 1 = excellent, 2 = very good, 3 = good, 4 = fair, 5 = poor.
**Vignette 2**	Gang Zhao/Li Wang has no problem walking 200 meters. However, after walking a mile or climbing several floors, he/she will feel tired. He/she can perform daily activities without assistance, such as buying food from markets and bringing it home. He/she has a headache each month, which will be alleviated after taking medicine. When he/she has a headache, he/she still can perform daily work. How would rate the health status of Gang Zhao/ Li Wang? Options: 1 = excellent, 2 = very good, 3 = good, 4 = fair, 5 = poor.

**Table 2:** All situations with two vignettes: this table gives calculations for the nonparametric estimator C for all possible situations with two vignette responses, v1 and v2, and a self-rated health response, y^a^.

Responses	y<v_1_	y = v_1_	v_1_<y<v_2_	y = v_2_	y>v_2_	C	Final value
**y<v**_**1**_ **<v**_**2**_	1	0	0	0	0	{1}	1
**y = v**_**1**_ **<v**_**2**_	0	1	0	0	0	{2}	2
**v**_**1**_ **<y<v**_**2**_	0	0	1	0	0	{3}	3
**v**_**1**_ **<y = v**_**2**_	0	0	0	1	0	{4}	4
**v**_**1**_ **<v**_**2**_ **<y**	0	0	0	0	1	{5}	5
**y<v**_**1**_ **= v**_**2**_	1	0	0	0	0	{1}	1
**y = v**_**1**_ **= v**_**2**_	0	1	0	1	0	{2,3,4}	/
**v**_**1**_ **= v**_**2**_ **<y**	0	0	0	0	1	{5}	5
**y<v**_**2**_ **<v**_**1**_	1	0	0	0	0	{1}	1
**y = v**_**2**_ **<v**_**1**_	1	0	0	1	0	{1,2,3,4}	/
**v**_**2**_ **<y<v**_**1**_	1	0	0	0	1	{1,2,3,4,5}	/
**v**_**2**_ **<y = v**_**1**_	0	1	0	0	1	{2,3,4,5}	/
**v**_**2**_ **<v**_**1**_ **<y**	0	0	0	0	1	{5}	5

^a^ The table was adapted from “King G, Wand J. Comparing Incomparable Survey Responses: Evaluating and Selecting Anchoring Vignettes. Political Analysis. 2007;15(1):46–66”. As shown in Table 1, vignette 1 described a person with better health than that in vignette 2. Thus, the response to vignette 1 (v_1_) was expected to be smaller than that to vignette 2 (v_2_). The situation of v1 being equal to v2 was called tied. The situation of v1 being bigger than v2 was called inconsistently ordered vignette response. In this analysis, we treated the tied values and inconsistently ordered vignette responses as missing values. Moreover, y represented the response to self-rated health question; ‘/’ represented missing value.

In all, 2,258 respondents (9.8%) did not supply valid answers to the vignettes, and 1,398 (6.1%) inconsistently ranked the ordering of vignette severity (i.e., assessed the health status in Vignette 1 as worse than that in Vignette 2). We treated these as missing values, which resulted in 3,656 (15.9%) respondents with missing values for adjusted SRH. Then we processed the missing values with Amelia II, a multiple imputation method based on the bootstrap used to impute incomplete data sets [43]. Using Amelia II, we generated five imputed complete data sets for the statistical analysis. For more details on anchoring vignettes see [35, 36] and on multiple imputation see [43, 44].

#### Social capital

Despite little agreement on the definition and measurement of social capital, trust is an essential component of SC [45] and the most frequently measured dimension of SC [3]. CFPS 2012 asked respondents to indicate their trust in different people. “If 0 represents the least trust and 10 the most, please score your trust of the following kinds of people”, including parents, neighbors, Americans, strangers, government officials, and doctors. According to their relevance to social capital, the trust of Americans was excluded. We used the average trust score for parents and neighbors to measure bonding SC, scores for strangers to measure bridging SC, and scores for government officials and doctors to measure linking SC. Bonding, bridging, and linking SC at the community level were measured as the average score of the corresponding SC at the individual level within each community.

#### Control variables

Demographic variables (sex, age, and marital status), socio-economic status (SES), and health risk factors were controlled in the multivariate analysis. SES variables included household registration (Hukou) type, migrant status, education (no more than elementary school, junior high, high school, and more than high school), employment, the natural logarithm of personal income (income measured in Yuan), and subjective SES (very low, low, medium, high, and very high). Health risk factors included health insurance, body mass index (BMI; kg/m^2^), smoking, and drinking. BMI was based on self-reported height and weight. According to the standard specifically designed for China, underweight was defined as BMI<18.5 and overweight or obese as BMI≥24; all other BMIs were considered normal weight [46]. Heavy smoking was defined as smoking at least 20 cigarettes per day; heavy drinking was defined as consuming at least 60 grams of pure alcohol per day for men and at least 40 grams for women. At the community level, we also controlled economic status through the natural logarithm of the average personal income within each community.

### Statistical analysis

We used descriptive statistics to depict characteristics of the sample. As suggested by Kawachi et al. [26], we used two-level ordinal logistic regression to model the relationship between SC and SRH at the individual and community levels. The multivariate analysis contained six models: Model 1 was a null model that included no explanatory variables. The intraclass correlation coefficient (ICC) and community-level variances were computed to examine the necessity of fitting two-level models [47]. Model 2 incorporated all control variables. Model 3 added individual-level social capital to Model 2. Model 4 added community-level social capital to Model 2. Model 5 added both individual-level and community-level social capital to Model 2. By comparing the results among Model 3, 4, and 5, we can see whether the relationship between individual-level social capital and adjusted SRH was influenced by the inclusion of community-level social capital and vice versa. Furthermore, given potential urban/rural disparities in the relationship between SC and SRH in China [28, 31], we included the interaction between residence and SC in Model 6. We also tested the interaction between SC at the individual and community levels. However, the effects were not significant and thus we did not include them in the final models. To analyze the impact of using anchoring vignettes on the association between SC and SRH, we ran two sets of Models 1–6 with adjusted SRH and SRH as outcome variables, respectively. Statistical analyses were conducted using Stata 13.1.

### Ethics statement

In this study, we did not collect data ourselves; instead we used an already published open-access database, which could be found on the CFPS official website (http://www.isss.edu.cn/cfps/). We were not able to access information which could be used to identify respondents. This study itself did not involve the issues of informed consent. The study complied with the Declaration of Helsinki and was reviewed and approved by the Institutional Review Board of Peking University Health Science Centre.

## Results

### Descriptive statistics

Of the three forms of SC, bonding SC had the highest score (7.77), followed by linking (5.96) and bridging SC (3.36; Table 3). Respondents’ average age was 44 years. Half of the respondents were male, 44% lived in urban areas, 79% were married, and 28% had urban Hukou. About 62% were middle class or higher. Nearly 60% had finished at least junior high school, the end of compulsory education in China. About 70% were employed, and the mean natural logarithm of personal income was 4.52. Moreover, 59% of respondents had normal BMI, 30% were overweight or obese, and 11% were underweight; and 16% and 4%, respectively, were heavy smokers and drinkers. Finally, 83% were covered by health insurance.

**Table 3:** Descriptive statistics.

Variable	Percent/ Mean(SE)	Variable	Percent/ Mean(SE)
**Social capital (SC)**		**Very high**	0.05(0.00)
** Bonding SC**	7.77(0.01)	**Education**	
** Bridging SC**	3.36(0.01)	**Lower than Junior high school**	0.41(0.00)
** Linking SC**	5.96(0.01)	**Junior high school**	0.33(0.00)
**Male**	0.50(0.00)	**High school**	0.16(0.00)
**Age**	44.02(0.11)	**Higher than high school**	0.10(0.00)
**Urban**	0.44(0.00)	**Employed**	0.69(0.00)
**Married**	0.79(0.00)	**Ln (personal income)**	4.52(0.03)
**Urban Hukou**	0.28(0.00)	**Body mass index**	
**Migrant**	0.11(0.00)	**Underweight**	0.11(0.00)
**Subjective social status**		**Normal**	0.59(0.00)
** Very low**	0.16(0.00)	**Overweight/obesity**	0.30(0.00)
** Low**	0.22(0.00)	**Heavy smoking**	0.16(0.00)
** Middle**	0.45(0.00)	**Heavy drinking**	0.04(0.00)
** High**	0.12(0.00)	**Health Insurance**	0.86(0.00)

### Self-rated health

**Fig 1:**
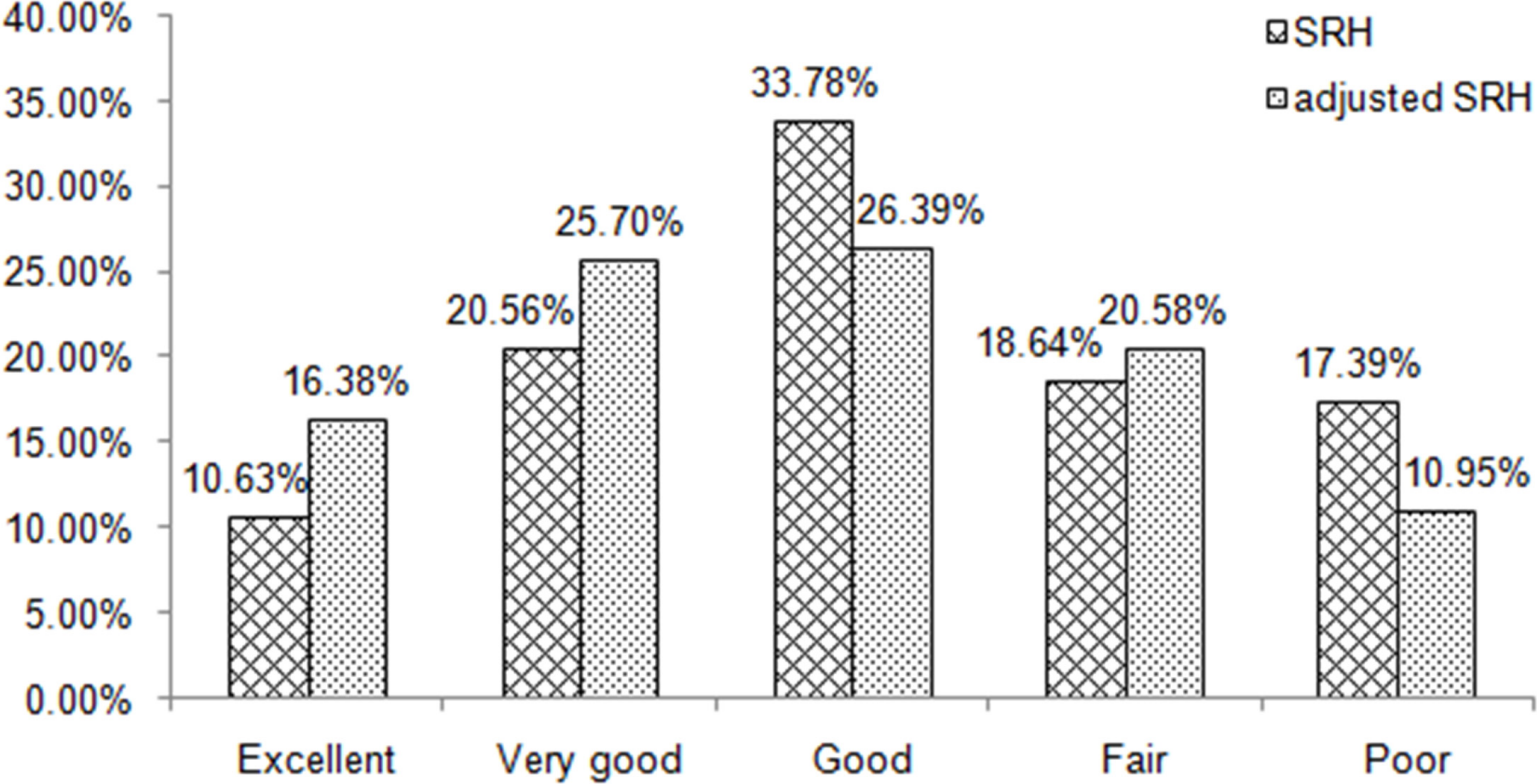
Distribution of self-rated health (SRH) before and after adjustment using anchoring vignettes.

As we showed in the Methods section, different respondents were expected to give the same answer to the same vignettes question, which described specific and fixed health traits. However, due to DIF, the answer to the same vignette question varied among respondents. For example, for Vignette 1 in Table 1, 22% of respondents chose excellent, 33% very good, 26% good, 6% fair, 4% poor, and 10% refused to answer. These variations reflected the interpersonal incomparability in SRH. When we used the vignettes technique to adjust SRH according to the calculation rule shown in Table 2, the five categories in SRH were transformed to the corresponding categories in adjusted SRH (Table 4). For example, among respondents reporting good health, only 43% remained the same after adjustment, 39% became excellent or very good, and 18% became fair or poor. During this process, the DIF in SRH was corrected in the new variable, i.e. adjusted SRH. These two measures were highly correlated (gamma = 0.8070, p<0.001), which implied the adjusted SRH still kept most of the distribution characteristics in SRH. Fig 1 displays the distribution of SRH before and after adjustment using anchoring vignettes. After adjustment, the distribution became flatter. The percent reporting good and poor health decreased from 34% to 26% and from 17% to 11%, respectively; all others increased.

**Table 4:** The relationship between self-rated health (SRH) before and after adjustment using anchoring vignettes.

SRH	Adjusted SRH					
	Excellent	Very good	Good	Fair	Poor	Total
**Excellent**	54.28%	45.72%	0.00%	0.00%	0.00%	100.00%
**Very good**	27.59%	47.71%	23.35%	1.08%	0.27%	100.00%
**Good**	7.86%	31.33%	42.75%	16.54%	1.52%	100.00%
**Fair**	2.98%	8.47%	45.61%	29.14%	13.79%	100.00%
**Poor**	1.41%	0.00%	0.00%	57.42%	41.17%	100.00%
**Total**	14.74%	26.49%	27.26%	21.13%	10.38%	100.00%

### Multivariate analyses

We performed six models to investigate the association of SC with adjusted SRH (Table 5). In the null model, the ICC was 0.041, which means 4.1% of the total variance in adjusted SRH could be explained by community-level variables such as social capital. And, the community-level variance (0.139) was significant, which also justifies the use of two-level regression. After we incorporated the control variables, the ICC declined from 0.041 to 0.040. Model 3 included individual-level SC. Persons with higher bonding and linking SC had a lower probability of reporting worse health (bonding SC, odds ratio [OR] = 0.97, 95% confidence interval [CI] 0.96–0.99; linking SC, OR = 0.97, 95% CI 0.95–0.98); however, bridging SC was marginally significantly and negatively related to adjusted SRH (OR = 1.02, 95% CI 1.00–1.03). In Model 4, community-level SC had no relationship with adjusted SRH. In Model 5, the associations between each SC variable and adjusted SRH were consistent with those in Models 3 and 4, except that community-level bridging SC had a marginally significant and positive association with adjusted SRH. Then, we examined the interaction effects between urban/rural residence and SC. In Model 6, at the individual level, such interaction effects were not significant and rendered bridging SC non-significant; at the community level, urban/rural disparities appeared in the association between bonding/linking SC and adjusted SRH. Residents in urban communities with higher bonding SC reported poorer health (OR = 1.22); however, the reverse was true in rural communities (OR = 0.89, 95% CI 0.79–1.02). In addition, higher community-level linking SC was related to better adjusted SRH among urban residents (OR = 0.89), but the opposite was true for rural residents (OR = 1.11, 95% CI 0.99–1.24). From Model 1 to 6, the reduced values of ICC from 0.041 to 0.038 indicated the models explained part of the community-level variance, which was consistent with the declining trend of community-level variance (0.139 to 0.130).

**Table 5:** Two-level ordinal logistic regression estimates (odds ratios and 95% confidence intervals) and variance components with adjusted self-rated health (SRH) as outcome variables.

Variable	Model 1	Model 2 ^a^	Model 3 ^a^	Model 4 ^a^	Model 5 ^a^	Model 6 ^a^
**Individual-level**						
** Bonding SC**			0.97(0.96–0.99)**		0.97(0.95–0.99)**	0.98(0.95–1.00)^+^
** Bridging SC**			1.02(1.00–1.03)^+^		1.02(1.00–1.04)^+^	1.02(0.99–1.04)
** Linking SC**			0.97(0.95–0.98)***		0.97(0.95–0.98)***	0.97(0.95–1.00)*
**Community-level**						
** Bonding SC**				1.00(0.90–1.10)	1.02(0.92–1.13)	0.89(0.79–1.02)^+^
** Bridging SC**				0.94(0.87–1.02)	0.93(0.86–1.00)^+^	0.90(0.82–0.99)*
** Linking SC**				0.98(0.89–1.07)	1.01(0.92–1.11)	1.11(0.99–1.24)^+^
**Individual-level**						
** Bonding SC** * **Urban**						0.99(0.95–1.03)
** Bridging SC** * **Urban**						1.00(0.96–1.04)
** Linking SC** * **Urban**						0.99(0.95–1.04)
**Community-level**						
** Bonding SC** * **Urban**						1.36(1.12–1.66)**
** Bridging SC** * **Urban**						1.09(0.92–1.29)
** Linking SC** * **Urban**						0.80(0.67–0.97)*
**Cut1**	-1.70(-1.75–1.64)***	-0.34(-1.06–0.37)	-0.70(-1.44–0.03)^+^	-0.67(-1.65–0.32)	-0.65(-1.64–0.34)	-1.13(-2.23–0.03)*
**Cut2**	-0.35(-0.40–0.30)***	1.11(0.39–1.84) ***	0.76(0.01–1.50)^+^	0.79(-0.20–1.78)	0.81(-0.18–1.80)	0.33(-0.77–1.43)
**Cut3**	0.78(0.74–0.83)***	2.39(1.66–3.11)***	2.03(1.28–2.78)***	2.06(1.07–3.05)***	2.08(1.09–3.07)***	1.60(0.50–2.70)**
**Cut4**	2.13(2.07–2.19)***	3.87(3.15–4.60)***	3.52(2.77–4.27)***	3.55(2.56–4.54)***	3.57(2.57–4.57)***	3.09(1.99–4.19)***
**Variance Components**						
**Community-level variance**	0.139***	0.137***	0.137***	0.135***	0.136***	0.130***
**Intra-class correlation**	0.041	0.040	0.040	0.039	0.040	0.038

^a^At the individual level, these models adjusted demographic variables (including sex, age and marital status), socio-economic variables (including Hukou, migrant status, educational attainment, employment, natural logarithm of personal income, subjective socio-economic status) and health risk factors (including health insurance, Body Mass Index, heavy smoking and heavy drinking); at the community level, they adjusted community economic status.

^+^ p<0.10;

^*^ p<0.05;

^**^ p<0.01;

^***^ p<0.001.

SC referred to social capital.

We also conducted the analyses using SRH as the outcome variable (Table 6). The ICC of Model 1 in Table 6 was 0.069, higher than that for adjusted SRH in Table 5 (0.041). The community-level variance of Model 1 in Table 6 (0.243) was also higher than that in Table 5 (0.139). Individual-level results for Model 6 were similar, but community-level results were quite different. There was an urban/rural disparity in the relationship between community-level bonding SC and SRH. Compared to the results in Table 5, in urban areas, the association between community-level bonding SC and SRH vanished (OR = 1.00); in rural areas, residents of communities with higher bonding SC were much more likely to report being healthy (OR = 0.66, 95% CI 0.57–0.77). Neither bridging/linking SC nor their interactions with urban or rural residency were significantly associated with SRH. In addition, as more variables, especially community-level variables were included in models, the values of ICC generally decreased from Model 1 to 6.

**Table 6:** Two-level ordinal logistic regression estimates (odds ratios and 95% confidence intervals) and variance components with self-rated health (SRH) as outcome variables.

Variable	Model 1	Model 2^a^	Model 3 ^a^	Model 4 ^a^	Model 5^a^	Model 6 ^a^
**Individual-level**						
** Bonding SC**			0.95(0.94–0.97)***		0.96(0.94–0.98)***	0.96(0.94–0.99)**
** Bridging SC**			1.02(1.01–1.04)*		1.02(1.00–1.04)*	1.01(0.99–1.04)
** Linking SC**			0.95(0.93–0.96)***		0.95(0.93–0.96)***	0.96(0.94–0.98)***
**Community-level**						
** Bonding SC**				0.75(0.67–0.85)***	0.78(0.69–0.88)***	0.66(0.57–0.77)***
** Bridging SC**				1.04(0.95–1.15)	1.02(0.93–1.13)	1.06(0.94–1.19)
** Linking SC**				1.02(0.92–1.14)	1.08(0.97–1.20)	1.12(0.97–1.28)
**Urban** * **Individual-level**						
** Bonding SC**						0.99(0.95–1.03)
** Bridging SC**						1.02(0.96–1.08)
** Linking SC**						0.96(0.93–1.00)
**Urban** * **Community-level**						
** Bonding SC**						1.52(1.21–1.92)***
** Bridging SC**						0.89(0.73–1.09)
** Linking SC**						0.95(0.77–1.17)
**Cut1**	-2.28(-2.35–2.21)***	-0.22(-1.04–0.59)	-0.85(-1.67–0.04)*	-1.96(-3.12–0.70)***	-1.93(-3.10–0.70)***	-2.80(-4.09–1.50)***
**Cut2**	-0.88(-0.94–0.82)***	1.31(0.50–2.12)***	0.68(-0.13–1.50)	-0.43(-1.159–0.70)	-0.40(-1.56–0.70)	-1.26(-2.55–0.04)^+^
**Cut3**	0.58(0.52–0.63)***	2.97(2.16–3.78)***	2.35(1.53–3.17)***	1.23(0.07–2.30)*	1.27(0.11–2.40)*	0.41(-0.89–1.70)*
**Cut4**	1.60(1.54–1.66)***	4.13(3.32–4.95)***	3.52(2.70–4.33)***	2.40(1.24–3.50)***	2.44(1.27–3.60)***	1.58(0.28–2.87)*
**Variance Components**						
**Community-level variance**	0.243***	0.253***	0.248***	0.234***	0.236***	0.225***
**Intra-class correlation**	0.069	0.071	0.070	0.066	0.067	0.064

^a^At the individual level, these models adjusted demographic variables (including sex, age and marital status), socio-economic variables (including Hukou, migrant status, educational attainment, employment, natural logarithm of personal income, subjective socio-economic status) and health risk factors (including health insurance, Body Mass Index, heavy smoking and heavy drinking); at the community level, they adjusted community economic status.

^+^ p<0.10;

^*^ p<0.05;

^**^ p<0.01;

^***^ p<0.001.

SC referred to social capital.

## Discussion

This paper mainly contributes to existing studies in two ways. It is among the first to assess the relationship between bonding, bridging, and linking SC and SRH [15, 18, 29]. Moreover, it is the first to use the anchoring vignettes technique to considerably improve the comparability of SRH [36, 37], which helps us understand the association of social capital with SRH more accurately and appropriately.

SC has mixed relationships with adjusted SRH among Chinese adults. First, individual-level bonding SC was positively related to adjusted SRH in urban and rural areas; community-level bonding SC was positively associated with adjusted SRH in rural areas, but negatively in urban areas. These findings are basically consistent with previous studies in China [28, 31]. Social support might be the main mechanism linking bonding SC and adjusted SRH, which is further complicated by the different social context in urban and rural China [18, 26, 48]. Social support could be related to health though multiple ways, such as through emotional, instrumental, or informational support, or through social companionship [3, 17, 27, 29, 49]. Furthermore, the association of social support with adjusted SRH may be either direct (e.g. provision of health information) or indirect (e.g. help with job search, which in turn promotes health). The situation of community-level bonding SC is more complex. Social relationships have dramatically changed in urban China alongside the development of a market economy; relationships between individuals with diverse social identities have become more important components of everyday life [48]. Accordingly, an urban community overemphasizing bonding SC probably compromises residents’ health by preventing them from obtaining health-related resources from other social groups [26, 45]. However, in rural China, traditional networks made of relatives and neighbors still dominate social life, which probably makes communities with abundant bonding SC a protective factor for health [28, 31].

Second, people living in communities with higher bridging SC tended to report better adjusted SRH in both urban and rural areas. In recent decades, social and economic inequality has been rising significantly in China [50, 51], which might lead to a negative impact on health through the socio-psycho-physiological mechanism illustrated by Wilkinson and colleagues [19, 52]. For example, socially disadvantaged people have a higher probability of suffering from long-term stress, which increases the chronic secretion of harmful levels of cortisol and adrenaline and in turn increases the probability of having diseases such as coronary heart disease and stomachaches [52]. Communities with higher bridging SC are more likely to establish trustful and respectful relationships between people with different social identities [15]. Such egalitarian relationships help to buffer the negative health impact brought by increased inequality [19, 20]. The results indicated no significant relationship between individual-level bridging SC and adjusted SRH in either urban or rural areas, which is inconsistent with our hypothesis that individual-level bridging SC is positively associated with SRH in urban areas. This hypothesis is based on our previous study using another nationwide survey dataset [31] and the different social relationships in urban and rural China, which have been discussed in detail in the last paragraph [48]. The inconsistency may come from at least two aspects: the level of bridging SC is low (Table 3) and the differences in bridging SC measurements.

Third, individuals with higher linking SC were more likely to report higher levels of health in both urban and rural areas; community-level linking SC was positively associated with adjusted SRH in urban areas, but negatively associated with adjusted SRH in rural areas. The higher linking SC indicated harmonious relationships between people across power and authority gradients [15, 53]. From the perspective of political economy, such relationships probably enable individuals to obtain more health-related resources, such as high-quality medical services [29]. Moreover, previous studies showed communities with higher linking SC may be more capable of negotiating with governments and authorities for health-related resources, and their residents are more likely to participate in community-based health interventions [18, 27]. These results provide some potential explanations for the positive relationships between community-level linking SC and adjusted SRH in urban China. However, in rural China, communities have been experiencing long-term underinvestment [48, 54, 55]. Even if some rural communities have higher linking SC, there are probably not so many health-related resources for them to mobilize. Furthermore, linking SC in poor rural areas could result in nepotism, corruption, and suppression, which negatively impacts overall population health [3, 15].

There is evidence of reporting heterogeneity or interpersonal incomparability in SRH. Respondents provided different responses to the same vignette questions, ranging from excellent to poor health, which verifies the necessity of using the anchoring vignettes technique to adjust SRH [36]. After DIF was reduced, the variation in SRH between communities decreased by more than 40% and the proportion of community-level variance in total variance measured as ICC in the null model became smaller. This is probably because people in the same community tend to have similarities in characteristics, such as, incomes, education, social norms, and health reference groups, which profoundly shape how they assess health [33, 34, 37]. The improved comparability of SRH affects the relationship between community-level SC and SRH: it revealed the negative association between bonding SC and SRH in urban areas; the reduced extent of the positive association between bonding SC and SRH in rural areas; and the mixed relationships between linking SC and SRH for urban and rural residents. Meanwhile, SRH comparability has little influence on the relationship between individual-level SC and health. Some factors that influence SRH reporting behavior are more homogeneous within the same community [34, 37]. Thus, when we improved the comparability of SRH and analyzed the data using two-level regression models, most of the changes were observed at the community level [32, 33].

Our study has two main limitations. First, the analysis was based on cross-sectional data, which makes us unable to exclude the possibility of mutual causality between SC and adjusted SRH. For example, it is possible that healthier persons have more energy to foster SC. Limited research has dealt with this problem using longitudinal data [56] or instrumental variables [27]. Second, we primarily measured SC using trust indicators. As described earlier, SC has three elements, i.e. social network, norms of reciprocity, and trust. CFPS 2012 did not include items on norms of reciprocity and only provided limited information on participation in organizations, which can’t be used to measure bonding, bridging, and linking social capital properly. Furthermore, according to previous Chinese studies, trust is the main social capital element associated with health; norms of reciprocity and social networks had little or even no relationship with population health [17, 28, 31]. We hope future studies have a better measurement of bonding, bridging, and linking SC.

## Conclusions

The distinction between bonding, bridging, and linking SC [15] helps us better understand the mechanism linking social capital and SRH using the social support, inequality and political economy perspectives. Our results indicated that the conclusion about the relationship between community-level SC and SRH might be distorted in analyses that do not take account of the interpersonal incomparability in SRH measurement, and thus we recommend that more studies use the anchoring vignettes technique to measure SRH. In addition, the significant urban/rural disparities in the association of SC with SRH reflect the need to consider the cultural and socioeconomic factors that characterize the environment when designing health intervention policies. Since this paper is conducted in China, which is an eastern and developing country, its conclusions should be generalized with caution.
